# LPS and its relationship with subjective–objective discrepancies of sleep onset latency in patients with psychiatric disorders

**DOI:** 10.1038/s41598-023-49261-4

**Published:** 2023-12-19

**Authors:** Keita Kawai, Kunihiro Iwamoto, Seiko Miyata, Ippei Okada, Motoo Ando, Hiroshige Fujishiro, Masahiko Ando, Akiko Noda, Norio Ozaki

**Affiliations:** 1 Department of Psychiatry, Nagoya University Graduate School of Medicine, 65 Tsurumai-Cho, Showa, Nagoya, Aichi 466-8550 Japan; 2 Department of Advanced Medicine, Nagoya University Hospital, Nagoya, Aichi Japan; 3 Department of Biomedical Sciences, Chubu University Graduate School of Life and Health Sciences, Kasugai, Aichi Japan; 4 Pathophysiology of Mental Disorders, Nagoya University Graduate School of Medicine, Nagoya, Aichi Japan

**Keywords:** Psychology, Diseases, Medical research

## Abstract

Subjective–objective discrepancies in sleep onset latency (SOL), which is often observed among psychiatric patients, is attributed partly to the definition of sleep onset. Recently, instead of SOL, latency to persistent sleep (LPS), which is defined as the duration from turning out the light to the first consecutive minutes of non-wakefulness, has been utilized in pharmacological studies. This study aimed to determine the non-awake time in LPS that is most consistent with subjective sleep onset among patients with psychiatric disorders. We calculated the length of non-awake time in 30-s segments from lights-out to 0.5–60 min. The root mean square error was then calculated to determine the most appropriate length. The analysis of 149 patients with psychiatric disorders showed that the optimal non-awake time in LPS was 12 min. On the other hands, when comorbid with moderate or severe obstructive sleep apnea (OSA), the optimal length was 19.5 min. This study indicates that 12 min should be the best fit for the LPS non-awake time in patients with psychiatric disorders. When there is comorbidity with OSA, however, a longer duration should be considered. Measuring LPS minimizes discrepancies in SOL and provides important clinical information.

## Introduction

Insomnia is one of the core symptoms of patients with psychiatric disorders^1^. Prolonged sleep onset latency (SOL) frequently occurs in various psychiatric disorders, with the residual insomnia symptoms including difficulty falling asleep, and having a negative impact on the treatment of psychiatric disorders^2,3^. Initiation of sleep involves a complex neural structures and neurotransmitters^4,5^. It begins with activation of the ventral lateral preoptic nucleus in the hypothalamus, which inhibits the arousal systems of the brainstem and forebrain^4^. Neurotransmitters involved include gamma-aminobutyric acid and galanin, which promote sleep, and histamine, norepinephrine, and serotonin, which are associated with wakefulness^5^. These mechanisms play an important role in sleep initiation, but subjective and objective sleep often diverge. Subjective and objective sleep assessments have been widely used to evaluate sleep, however, discrepancies between subjective and objective sleep durations are common in psychiatric patients, with this condition known as sleep state misperception^6–8^. Even when patients objectively get a regular amount of sleep, their sleep state misperception can cause worry and anxiety about sleep, thereby leading to an objective sleep disturbance^9^. Although the underlying mechanism of the sleep state misperception remains elusive, previous research has discovered several variables, such as cognitive and psychological factors, the ability to estimate time, physiological factors^9–11^.

In addition to the above, the definition and measurement of sleep onset could be related to the misperception of SOL^9^. According to the American Academy of Sleep Medicine (AASM), the definition of conventional SOL is the time from lights out to the start of the first epoch of any of the sleep stages (N1, N2, N3, rapid eye movement: REM)^12^. In contrast, a new concept of latency to persistent sleep (LPS) has been defined as the duration from turning out the light to the first consecutive minutes of non-wakefulness^13^. LPS is increasingly being used as a concept to measure SOL in developmental research on sleep drugs, as LPS has the advantage of being able to measure sleep persistence^13,14^. Since sleep in insomniacs is typically inconsistent, the assessment of continuous state of sleep onset for a defined period, such as the LPS, is likely to be more accurate than using the standard epoch measurements.

Although this LPS non-awake time is often set at 10 min, ranging from 5 to 34 min has been reported, no consistent findings have yet been obtained^15–20^. Furthermore, since most of these studies focused on primary insomnia, it has yet to be definitively determined as to how long this continuous length best reflects the subjective sleep onset in patients with psychiatric disorders, as these patients may have different sleep structures^21,22^. Therefore, this study attempted to find the most appropriate non-awake time in LPS among psychiatric patients in real-world clinical practice.

## Methods

### Participants

**Figure 1:**
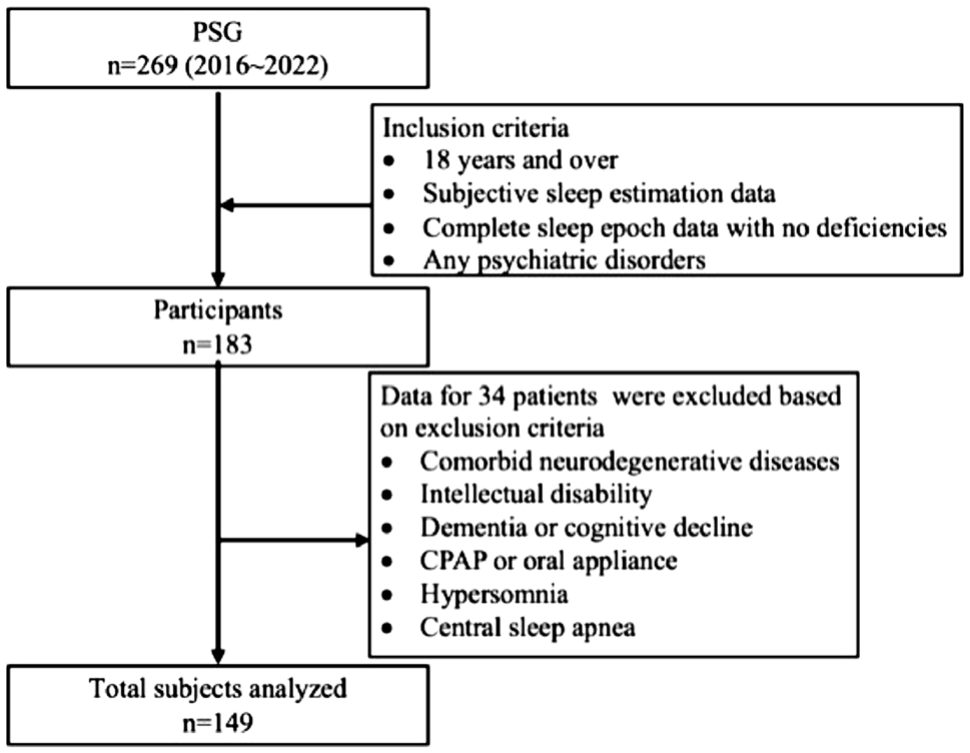
Procedure for recruitment of subjects. *PSG* polysomnography, *CPAP* continuous positive airway pressure.

We conducted a cross-sectional single-center study. This study analyzed the data from patients who had undergone polysomnography (PSG) at Nagoya University Hospital from 2016 to 2022. Inclusion criteria were: (1) 18 years of age or over, (2) availability of subjective sleep estimation data, (3) complete sleep epoch data with no deficiencies, and (4) the patient met any of the criteria for psychiatric disorders based on DSM-5. Exclusion criteria were: (1) comorbid neurodegenerative diseases, (2) intellectual disability, (3) dementia or cognitive decline, (4) having undergone PSG while using a continuous positive airway pressure or oral appliance, (5) hypersomnia, and (6) central sleep apnea. Out of the 269 total patients, 149 patients met these criteria and were included in our analysis (Fig. 1). All diagnoses were made by several skilled psychiatrists and all participants had stables symptoms. Patients completed a self-administered questionnaire prior to performing the PSG, and estimated their subjective SOL in the morning after the PSG overnight. All procedures were approved by the Ethics Review Committees at Nagoya University Hospital. Written informed consent in compliance with the guidelines of the Declaration of Helsinki was provided by all patients and obtained prior to participation.

### Self-reported questionnaires

#### Pittsburgh Sleep Quality Index (PSQI)

The Pittsburgh Sleep Quality Index, which is a 19-item instrument that assesses subjective sleep quality, SOL, and total sleep time (TST), was used to assess sleep quality^23^. The questionnaire consists of seven elements, with a total score ranging from 0 to 21. Previous studies have reported that patients with a score of 6 or above were poor sleepers. In addition, we defined difficulty falling asleep based on the PSQI subscale as follows: SOL for question 2 was more than 30 min, while the answer for question 5a regarding the number of times a patient could not get to sleep being reported as “three or more times per week.

### Polysomnography

This investigation utilized a standard PSG (Embla N7000, Natus Neurology Incorporated, WI, USA or PSG-1100, Nihon Kohden Co., Tokyo, Japan). The AASM scoring manual, version 2.1, was used to assess the results^12^. Details for these techniques have been previously published^24^. Trained sleep technologists evaluated the TST, SOL, and apnea–hypopnea index (AHI). PSG recordings were consistently conducted over an 8-h period. Specifically, the recordings were carried out either from 9 p.m. to 5 a.m. or from 10 p.m. to 6 a.m.

### Subjective sleep onset latency

After completing the PSG in the morning, participants were asked to estimate their subjective SOL using the following question: *“How long did it take you to fall asleep?”*. Participants then provided their SOL in hours and minutes.

### Latency to persistent sleep

The duration of the first sleep epoch after turning out the lights and the specific sustained non-awake time were calculated in this study based on the data from each participant that were collected in 30 s increases, which ranged from 0.5 to 60 min of no interrupted sleep. If an awakening epoch was observed during the monitored sleep period, this sleep period was not considered to be continuous sleep^18^.

### Statistical analysis

All data except for age and score of PSQI are presented as the median and interquartile range (IQR). Root mean square error (RMSE) was calculated between the subjective SOL and each LPS with a non-awake time of 0.5–60 min. Then, we plotted the RMSE on a graph in order to detect the most suitable value for the sleep duration. A lower RMSE indicated that there was no difference between the two sleep measurements. All statistical analyses in this study were performed using R 4.1.1.

## Results

Table 1 presents the patient’s clinical characteristics. In total, 149 patients were evaluated (89 males, 60 females), with a mean age of 47.2 years (SD 16.8 years). The prevalence of the psychiatric disorders was as follows: major depressive disorders (*n* = 45, 30%), bipolar disorders (*n* = 34, 23%), neurodevelopmental disorders (*n* = 29, 19%), schizophrenia (*n* = 14, 9.4%), somatic disorders (*n* = 12, 8.1%), other disorders (*n* = 10, 6.7%), and anxiety disorders (*n* = 5, 3.4%). For sleep disorders, these included: obstructive sleep apnea (OSA) (*n* = 99, 64%), periodic limb movement disorder (PLMD) (*n* = 4, 2.7%), REM sleep behavior disorder (RBD) (*n* = 3, 2.0%), and restless legs syndrome (RLS) (*n* = 1, 0.7%). The prevalence of medication use among participants was as follows: benzodiazepines were the most commonly used (*n* = 64, 43.0%), followed by Antipsychotics (*n* = 59, 39.6%), Antidepressants (*n* = 55, 36.9%), Hypnotic (*n* = 45, 30.2%), Mood stabilizers (*n* = 31, 20.8%), and Anti-Parkinson drugs (*n* = 1, 0.7%).

**Table 1:** Summary of measured variables.

	Total n = 149
Demographics	
Age (years)	47.2 ± 16.8
Sex (female/male)	60/89
Sleep measures	
Subjective sleep (min)	
TST	360.0 (300.0–420.0)
SOL	30.0 (20.0–60.0)
Objective sleep (min)	
TST	398.0 (341.5–440.0)
SOL	10.5 (5.00–30.00)
LPS	22.5 (9.50–43.8)
WASO	94.0 (57.0–147.0)
Sleep stages	
REM (%TST)	14.6 (8.90–18.8)
N1 (%TST)	30.4 (22.3–47.00)
N2 (%TST)	50.5 (37.4–58.7)
N3 (%TST)	0.00 (0.00–2.60)
AHI	10.7 (3.90–24.7)
PSQI	9.3 ± 4.2
Psychiatric diagnostic classification, number (%)	149 (100)
Major depressive disorders	45 (30)
Bipolar disorders	34 (23)
Neurodevelopmental disorders	29 (19)
Schizophrenia	14 (9.4)
Somatic disorders	12 (8.1)
Other disorders	10 (6.7)
Anxiety disorders	5 (3.4)
Sleep disorders, number (%)	107(100)
OSA^a^	99 (64)
PLMD	4 (2.7)
RBD	3 (2.0)
RLS	1 (0.7)
Medication, number (%)^b^	
Benzodiazepines	64 (43.0)
Antipsychotics	59 (39.6)
Antidepressants	55 (36.9)
Hypnotic	45 (30.2)
Mood stabilizers	31 (20.8)
Anti-Parkinson drugs	1 (0.7%)

*TST* total sleep time, *SOL* sleep onset latency, *AHI* apnea hypopnea index, *PSQI* Pittsburgh Sleep Quality Index, *OSA* obstructive sleep apnea, *PLMD* periodic limb movement disorders, *RBD* rapid eye movement sleep behavior disorders, *RLS* restless legs syndrome, *PLMI* periodic leg movement during sleep index.

^a^OSA is defined by an AHI of ≧ 5 events/h. PLMD is defined by PLMI ≧ 15.

^b^The percentages in the table reflect the prevalence of each drug category among the study participants. It is important to note that the totals exceed 100% as individuals were prescribed medication from multiple categories.

**Figure 2:**
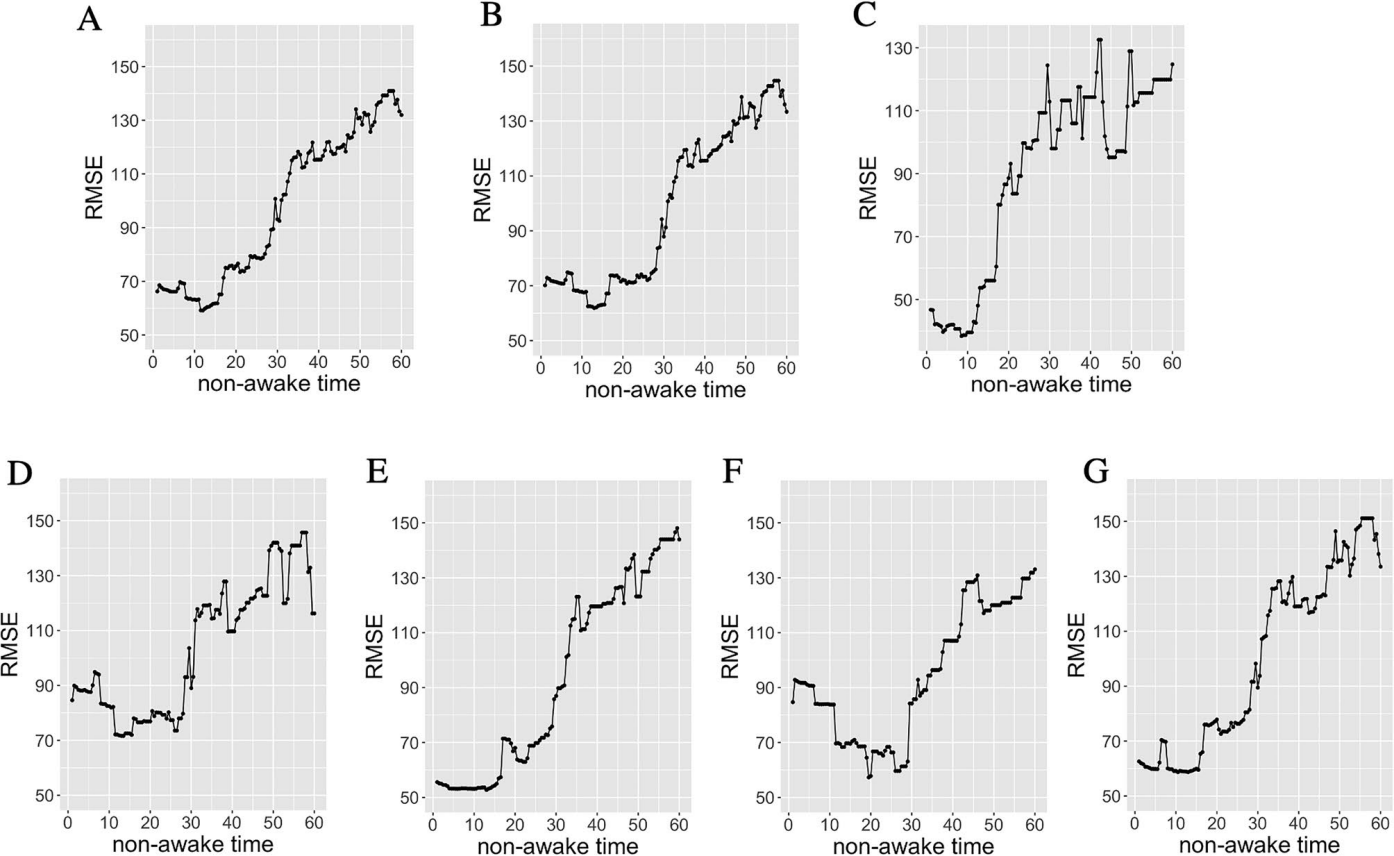
The RMSE outcomes between subjective SOL and LPS. The x-axis represents the length of non-awake time (min). The y-axis represents the RMSE. (**A**) For all participants (n = 149), the optimal length of non-awake time was 12 min. (**B**) For participants with subjective insomnia (n = 120), the optimal length of non-awake time was 13 min. (**C**) For participants without subjective insomnia (n = 29), the optimal length of non-awake time was 8.5 min. (**D**) For participants with subjective insomnia and difficulty in falling asleep (n = 54), the optimal length of non-awake time was 13–13.5 min. (**E**) For participants with subjective insomnia but no difficulty in falling asleep (n = 66), the optimal length of non-awake time was 13 min. (**F**) For participants with subjective insomnia and moderate or severe OSA (n = 37), the optimal length of non-awake time was 19.5 min. (**G**) For participants with subjective insomnia and no OSA (n = 83), the optimal length of non-awake time was 10 min. *RMSE* root mean square error, *SOL* sleep onset latency, *OSA* obstructive sleep apnea, *TST* total sleep time, *SOL* sleep onset latency, *WASO* wake after sleep onset, *AHI* apnea hypopnea index, *PSQI* Pittsburgh Sleep Quality Index, *OSA* obstructive sleep apnea, *PLMD* periodic limb movement disorders, *RBD* rapid eye movement sleep behavior disorders, *RLS* restless legs syndrome, *PLMI* periodic leg movement during sleep index, *PSG* polysomnography, *CPAP* continuous positive airway pressure, *RMSE* root mean square error, *REM* rapid eye movement, *LPS* latency to persistent sleep, *IQR* interquartile range.

LPS was calculated based on each non-awake time, with the RMSEs calculated from the subjective SOL and each LPS with a non-awake time of 0.5–60 min in order to identify the optimal sleep duration for the latency (Fig. 2). An RMSE of 12 min of sleep duration was the lowest value found for all of the psychiatric patients’ data (Fig. 2A, n = 149). Based on these data, we then calculated the RMSEs for those patients who reported having subjective insomnia symptoms and those who did not, respectively, as indicated by the PSQI. Figure 2B (*n* = 120) indicated that those with subjective insomnia had the lowest RMSE at 13 min, while Fig. 2C (*n* = 29) shows that the lowest RMSE in patients without subjective insomnia was 8.5 min. After adding the symptoms of difficulty falling asleep, the lowest RMSE for those patients suffering from this issue was 13–13.5 min as compared to 13 min for those who did not have this issue (Fig. 2D, n = 54, Fig. 2E, n = 66). We also calculated the RMSE for subjective insomnia symptoms combined with moderate or severe OSA The RMSE was smallest at 19.5 min when insomnia symptoms were comorbid with moderate or severe OSA, and 10.5 min when no OSA was comorbid (Fig. 2F, n = 37, Fig. 2G, n = 83).

## Discussion

The aim of this study was to determine the most appropriate non-awake time that is concordant with the subjective SOL in patients with psychiatric disorders. Our results revealed that among all of the psychiatric patients, the duration from the lights out to 12 uninterrupted minutes of LPS non-awake time was an optimal length. In addition, among psychiatric patients with moderate or severe OSA, 19.5 LPS non-awake time was the most optimal. These findings demonstrate the importance of measuring LPS as a way to minimize overestimation of SOL.

The 12-min length was roughly approximate to the current duration of LPS non-awake time that is used in clinical settings. Carskadon^19^ proposed the first consecutive 10-min sleep epoch latency that occurs from lights out to falling asleep. A previous study reported that the concordance between the subjective and objective SOL was optimal when the first epoch of N2 lasted for at least 15 min^20^. However, other suggested 34 and 22 min as the optimal non-wakefulness for insomniac and normal subjects, respectively^18^. One possible reason for this discrepancy is that the original subjective and objective SOL in our participants were shorter than that observed for the other studies. The median subjective and objective SOL for insomniacs was 123 and 25 min, respectively, whereas our median subjective and objective SOL for psychiatric patients was 30 and 10.5 min, respectively. Even among patients with psychiatric disorders who reported subjective insomnia, the median subjective SOL was 42.5 min while the median objective SOL was 11.75 min. Thus, differences found in the SOL of our sample may have led to the differences observed from the previous studies.

Furthermore, we demonstrated that the existence of OSA and its severity may prolong the LPS non-awake time. Previous studies have reported that the OAS was associated with SOL overestimation. In other words, patients with OSA have shorter objective SOL and longer subjective SOL^25^. A possible mechanism for this could be associated with the relationship found between sleepiness and the OSA. Results of studies that have investigated SOL and sleepiness in OSA patients suggested that higher daytime sleepiness was associated with shorter objective SOL^26,27^. These results suggested that the high levels of daytime sleepiness observed in patients with OSA increases sleep pressure and may have shorten the time it takes to fall asleep. Moreover, patients with OSA have been reported to have more awakenings during sleep, with more unstable sleep stages observed as compared to patients without OSA^28,29^. In other words, it's possible that people with OSA fall asleep faster due to their daily sleepiness, but they experience awaking after they fall asleep. Based on these previous findings, the current sleep index, which is the duration between the turning out of the lights and the onset of a particular stage of sleep as measured in a 30-s epoch, may ignore the awakenings that occur after judgement of falling sleep. Furthermore, one experimental study reported finding that by suppressing breathing during sleep onset, participants were not aware of the subjective falling asleep, even if the stageN2 condition lasted for 25 min^30^. Therefore, this widely used optimal length for sleep onset decisions appears to be longer than the ten consecutive minutes currently being used.

There are several strengths of our study. First, to the best of our knowledge, this is the first study to have investigated the LPS non-awake time among patients with psychiatric disorders. Second, the length that we define in our study takes into consideration the comorbidity with OSA, which is found in a high prevalence of psychiatric patients^31^.

However, there were also several limitations of the present study. First, the subgroup analysis for each of the psychiatric disorders was impossible to carry out due to the small sample size of this study. Furthermore, we were unable to establish a control group. Second, the results of our study were not completely adjusted for the effects of psychiatric drugs, as many of the participants took multiple drugs, not just one drug. Since previous studies have reported on some of the effects of psychiatric drugs on sleep^32–34^, it will be necessary to further investigate how the type of drug and dosage can affect the findings of this type of research. Third, the measurements of the sleep of all participants were collected under controlled conditions. Thus, these results should be viewed with some caution, as measurements of sleep when obtained in home settings could very well be different from those obtained in a laboratory setting. Finally, we did not collect the data on their habit of nap. Taking nap can deprive sleep pressure and it would influence on sleep onset latency, although short naps do not have much of an effect^35,36^. Future research will need to examine the relationship between these findings and additional indices, including psychological and symptom rating scales while considering other confounders such as usual sleep habits and gender difference.

In conclusion, the appropriate LPS non-awake time among psychiatric patients was 12 min, which was not largely different from the existing length of 10-min LPS currently being utilized in other settings. However, the length of consecutive sleep for the purpose of calculating a sleep score can be longer for subjects that are comorbid with moderate or severe OSA. These findings can facilitate more accurate sleep assessment and appropriate treatment decisions in actual clinical practice, and may be one of the factors that minimize misperception of SOL.

## Data Availability

The datasets used and/or analyzed during the current study available from the corresponding author on reasonable request.
